# Supplementary material for: Prevalence of antibiotic-resistant Escherichia coli isolates from healthy chicken droppings
Source: PLoS One. 2025 Dec 4;20(12):e0337055. doi: 10.1371/journal.pone.0337055 (PMC12677439; doi:10.1371/journal.pone.0337055)
Supplement: Attachment — Submitted filename: Review Comments to the Author.docx [file pone.0337055.s001.docx]

Review Comments to the Author:

The authors have addressed a significant public health issue—the prevalence and molecular characteristics of antibiotic-resistant E. coli in poultry, comparing broiler and indigenous chickens in Kifri City, Sulaymaniyah Governorate, Kurdistan Region, Iraq. The topic is highly relevant, timely, and aligns well with global concerns regarding antimicrobial resistance (AMR) and the One Health approach. The manuscript is generally well-structured, with comprehensive methodology and appropriate statistical analyses. I have no hesitation recommending this paper for publication, but I suggest revisions to improve clarity and scientific rigor:

**Introduction:**

Please consider including recent references beyond 2022 to reinforce the current relevance of your research and demonstrate awareness of the latest developments in this rapidly evolving field.

**Materials and Methods:**

The methodology of this study appears straightforward and easy to follow, which enhances its reproducibility and applicability for similar future research. The study design and procedures are clearly described, making it accessible for other researchers to replicate or build upon. However, some limitations should be acknowledged: the sample size seems relatively small for a survey. Additionally, please specify the number of farms involved in the study. For example, clarify whether the 100 samples originated from a single farm, multiple farms, or different locations within Kifri City. This information is essential for understanding the scope and representativeness of your findings.

**Results:**

Figures are not referenced within the text, and they appear to replicate information already presented in the tables. Please ensure all figures are properly cited in the manuscript and contribute additional value or clarity to the results.

**Molecular Detection of Resistance Genes**:

Authors should consider linking the presence of resistance genes with phenotypic resistance patterns, possibly through correlation analysis. This would strengthen the interpretation of the molecular findings and their practical implications.

**Discussion:**

If the sample size is limited, explicitly acknowledge this in the discussion. Address how it might affect the generalizability and representativeness of your findings, and suggest that further studies with larger, more diverse samples are needed.

Additionally, discuss potential limitations such as sampling bias, regional specificity, or the lack of genomic typing, and how these factors might influence your results.
