# Supplementary material for: Prevalence of antibiotic-resistant Escherichia coli isolates from healthy chicken droppings
Source: PLoS One. 2025 Dec 4;20(12):e0337055. doi: 10.1371/journal.pone.0337055 (PMC12677439; doi:10.1371/journal.pone.0337055)
Supplement: Attachment — Submitted filename: Review.docx [file pone.0337055.s002.docx]

The manuscript addresses a highly relevant topic: antimicrobial resistance (AMR) in *Escherichia coli* isolated from poultry under different farming systems in Iraq. The study design is appropriate, the methodological framework follows standard guidelines, and the discussion effectively connects the findings to the One Health perspective and the Sustainable Development Goals (SDGs). Once the comments outlined below are addressed, the manuscript will constitute a valuable contribution to the literature.

In the Introduction, the first schematic figure contains a typographical error. It should read: *“Isolate Escherichia coli.”*

In the Materials and Methods section, under the subsection *Molecular Detection of Resistance Genes*, it should be specified whether DNA extraction and subsequent PCR were performed on all 200 swabs. At present, it is unclear whether all samples were analyzed or only a subset.

In the Materials and Methods section, under the subsection *Statistical Analysis*, it is stated that 95% confidence intervals are reported; however, these are not presented in the Results section.

In the Materials and Methods section, under the subsection *Ethics Statement*, the approval date (23 December 2024) is later than the sampling period (December 2022 to April 2023), which requires clarification.

Additionally, reported percentages in the text do not always match those presented in the tables (Table 2). For example:

- Amoxicillin (broilers): text reports 97.5%, Table 2 shows 96.7%.
- Amoxicillin–clavulanic acid (broilers): text reports 97.5%, Table 2 shows 95.0%.
- Norfloxacin (broilers): text reports 92.5%, Table 2 shows 91.7%.

Finally, in Table 3, explanatory notes such as *“Approach to Assigning p-values”* and *“Hypothetical Example”* are included. These are inappropriate in a research article and must either be removed or transferred to the supplementary materials.
