# Supplementary material for: Prevalence of antibiotic-resistant Escherichia coli isolates from healthy chicken droppings
Source: PLoS One. 2025 Dec 4;20(12):e0337055. doi: 10.1371/journal.pone.0337055 (PMC12677439; doi:10.1371/journal.pone.0337055)
Supplement: Attachment — Submitted filename: Reviewers Response.docx [file pone.0337055.s004.docx]

**Response: Reviewer 1**

We appreciate the reviewer’s valuable comment. DNA extraction and PCR analysis were not performed on all 200 swab samples, as conducting molecular testing on the entire collection would have been prohibitively expensive and time-consuming. Instead, PCR was carried out on representative isolates that were phenotypically resistant, selected based on their antibiotic resistance profiles. This approach allowed us to focus on detecting key resistance genes among the most relevant isolates.

We thank you for this helpful observation. We acknowledge that the 95% confidence intervals (CIs) were mentioned in the Statistical Analysis section, but were not presented in the Results. The manuscript has been revised accordingly, and the 95% CIs are now included in the text of the Results section to provide a more precise representation of data precision.

The approval date (23 December 2024) reflects the official documentation issued by the Institutional Animal Care and Use Committee (IACUC) for the ongoing research project under which the sampling activities were conducted. The sampling period (December 2022–April 2023) was carried out as part of this approved research framework, and all procedures complied with institutional ethical standards.

We thank you for pointing out the discrepancies between the text and Table 2. The percentages reported in the text have now been corrected to match the values presented in Table 2. All data have been carefully checked to ensure consistency throughout the manuscript.

The explanatory notes in Table 3, including “Approach to Assigning p-values” and “Hypothetical Example,” have been removed from the main table to comply with journal standards. Any necessary explanatory information has been transferred to the supplementary materials.

**Response: Reviewer 2**

We appreciate your observation. To strengthen the introduction and provide recent evidence supporting the statement regarding the emergence and dissemination of multidrug-resistant *Escherichia coli* (MDR *E. coli*), we have added an updated reference (Leclercq et al., 2024) at the end of the first paragraph in the Introduction.

We thank you for this valuable observation. In response, we have clarified in the revised manuscript that the 100 samples were collected from multiple farms located in different areas within Kifri City to enhance representativeness and reduce sampling bias. Although the overall sample size may be considered moderate, the inclusion of samples from several independent farms strengthens the diversity and reliability of the findings, and we clarified this in the revised version.

We thank you for this valuable comment. All figures in the manuscript were originally generated by the authors and not taken from external sources.

In the revised manuscript, we have added a correlation analysis linking the presence of resistance genes with the corresponding phenotypic resistance patterns. The results showed a clear association between genotypic and phenotypic resistance among both broiler and indigenous isolates. Specifically, blaTEM, qnrS, and sul1 genes were more prevalent in broiler isolates and were consistent with resistance to β-lactams, quinolones, and sulfonamides, respectively.

The Discussion section has been revised to acknowledge the study’s limitations, including the relatively small sample size, potential sampling bias, and regional specificity. We also noted the lack of genomic typing as a limitation that may affect the depth of genetic analysis. A statement has been added suggesting that future studies with larger and more diverse samples, supported by genomic tools, are needed to confirm and extend our findings.
